# Iatrogenic injury to an unusual anatomic variant of the internal thoracic artery: lateral costal artery

**DOI:** 10.1186/s42155-026-00685-9

**Published:** 2026-05-08

**Authors:** Zhonghao Cui, David S. Shin, Jonathan C. Giang, Jeffrey Forris Beecham Chick, Ben Varughese, John Weaver, Mina S. Makary

**Affiliations:** 1 Section of Vascular and Interventional Radiology, Department of Radiology, University of Washington, 1959 Northeast Pacific Street, Seattle, WA 98195 USA; 2 Northeast Ohio Medical University, Rootstown, OH 44272 USA; 3 Division of Vascular and Interventional Radiology, Department of Radiology, The Ohio State University Wexner Medical Center, Columbus, OH 43210 USA

To the Editor

The lateral costal artery (LCA) represents an underrecognized but clinically relevant anatomic variant of the internal thoracic artery (ITA). It occurs in approximately 11–19% of individuals and typically arises from the proximal ITA, usually 2–3 cm below the subclavian artery, and travels laterally along the anterior chest wall [1, 2]. The LCA extends a variable distance along the chest wall, most commonly spanning two intercostal spaces, though it can extend as far as the fourth to seventh intercostal spaces in some cases [2]. The vessel is most often unilateral (55% of cases when present) but can occur bilaterally [3]. The LCA can be identified preoperatively using computed tomography (CT) angiography or intraoperatively with indocyanine green fluorescence. It anastomoses with intercostal arteries and may function as a collateral blood supply circuit for the thoracic wall [1]. Surgeons and interventionalists performing thoracic procedures should be aware of this variant to avoid complications. We present a case in which an aberrant lateral costal artery had significant implications for the angiographic management of thoracic bleeding.

A 33-year-old male presented with 1 month of night sweats and 2 weeks of pressure sensation in the right chest. Subsequent work-up with CT revealed a 4.3 × 2.2 × 3.5-cm pleural mass in the lateral right hemithorax. Seventeen days later, percutaneous biopsy of the mass using CT guidance and coaxial technique was performed to obtain two 18-gauge core tissue samples. A semi-automated biopsy device was used. Pathologic differential diagnoses included spindle cell carcinoma, melanoma, and carcinomatous sarcoma.

Thirty-one days after the biopsy, the patient was found unresponsive by family. He was transferred to the hospital by ambulance. The patient was hypotensive and tachycardic on arrival. Initial laboratory evaluation showed hemoglobin of 7.6 g/dL (normal reference range 13–18 g/dL). CT angiography of the chest showed a large right hemothorax with a variant lateral branch of the ITA coursing along the pleural base of the tumor (Fig. 1).

**Fig. 1:**
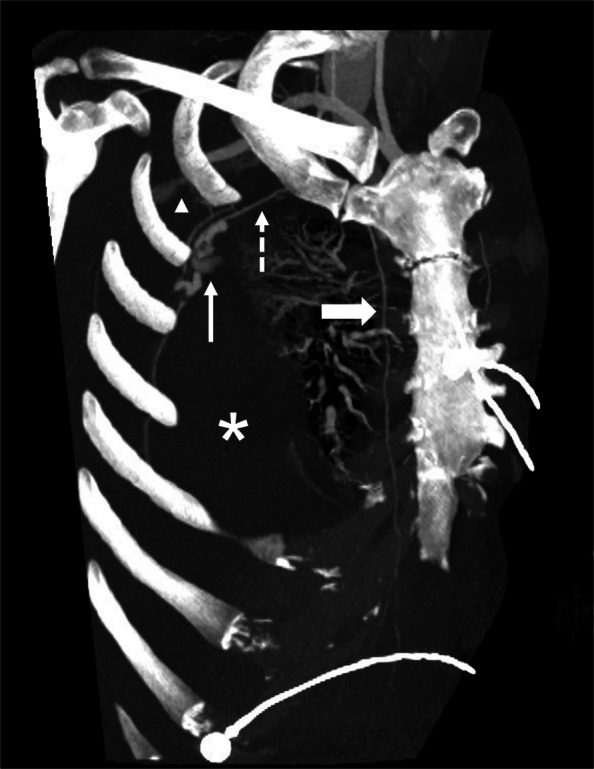
Multiplanar maximum intensity projection reformat image of the chest computed tomography demonstrating the internal thoracic artery (thick solid arrow), lateral costal artery (dashed arrow), intratumoral pseudoaneurysms (thin solid arrow), early filling axillary vein (arrowhead), and hemothorax (asterisk)

The patient was emergently brought to an angiography suite. Right radial artery access was obtained, and a 5-French sheath was placed. A 5-French angled catheter (Glidecath, Terumo; Tokyo, Japan) was used to select the right ITA origin, and arteriography demonstrated the variant lateral branch with intratumoral pseudoaneurysms and arteriovenous fistulae along its course (Fig. 2). A 2.4-French microcatheter (Progreat, Terumo; Tokyo, Japan) was advanced along the lateral branch distal to the vascular lesions. Multiple detachable microcoils (Embold, Boston Scientific; Marlborough, MA, USA) were deployed to embolize a long segment of the lateral branch were deployed to embolize a long segment of the lateral branch (Fig. 3). Post-embolization arteriography showed no residual pseudoaneurysm or arteriovenous fistula. The microcoils used were two 2 mm x 80 mm microcoils, three 4 mm x 30 mm microcoils, and two 5 mm x 30 mm microcoils. These microcoils were deployed in a distal-to-proximal (sandwich) approach to occlude distal outflow first, thereby preventing retrograde reperfusion via collateral pathways before proximal inflow control was established. A proximal-only approach would plug the vessel proximally, while distal collaterals can still re-perfuse. A distal embolization is recommended to avoid reperfusion from collaterals if a proximal-only approach was utilized. Alternative embolic agents may be considered depending on vessel tortuosity and the presence of collateral supply. Liquid embolics such as n-butyl cyanoacrylate (NBCA) allow for distal penetration and rapid occlusion, but their use is limited by reduced control and the potential for non-target embolization. Conformable embolics (Obsidio, Boston Scientific; Marlborough, MA, USA) provide a cohesive embolic column with minimal fragmentation and improved delivery control, though their performance in small, tortuous thoracic arterial variants remains incompletely defined [4]. In this case, detachable microcoils were selected to allow controlled, segmental occlusion of the lateral costal artery while minimizing unintended embolization.

**Fig. 2:**
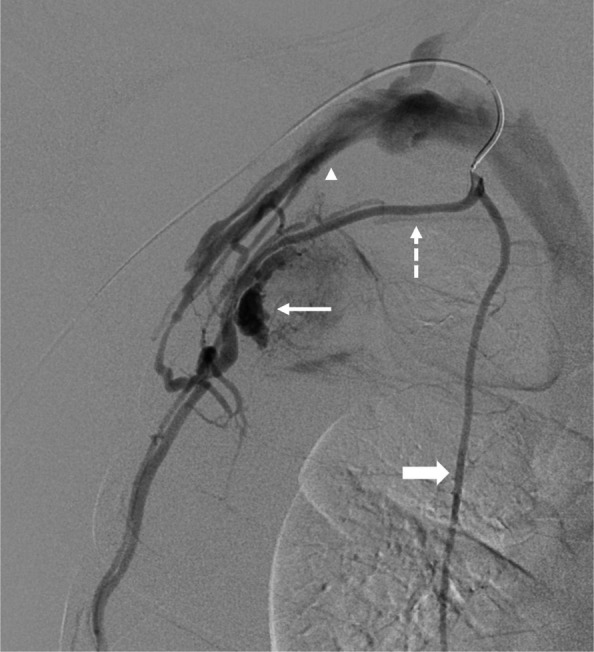
Selective digital subtraction arteriography of the right internal thoracic artery (thick solid arrow) demonstrating an early lateral branch (lateral costal artery; dashed arrow), which courses across the pleural mass and continues distally to traverse multiple intercostal spaces. Intratumoral pseudoaneurysms (thin solid arrow) are noted. Multiple channels of early draining veins connect to the right axillary vein (arrowhead), consistent with arteriovenous fistulae

**Fig. 3:**
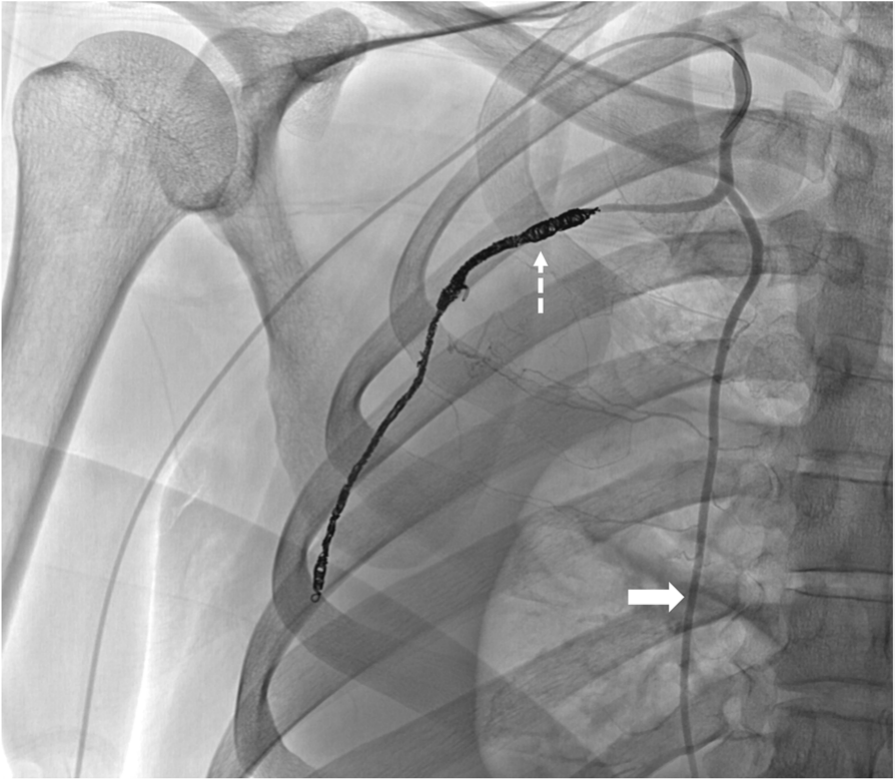
Postembolization arteriography of the right internal thoracic artery (thick solid arrow) demonstrating a coil pack along the lateral costal artery (dashed arrow) with no residual pseudoaneurysm or arteriovenous fistula

Over the next 2 days, the patient remained hemodynamically stable with no ongoing need for transfusion. Two days after the angioembolization, he underwent a right video-assisted thoracoscopic surgery (VATS) for hematoma evacuation. He was discharged on hospital day 6.

The ITA demonstrates several anatomic variants [2, 3]. An aberrant lateral branch arising from the proximal ITA has been described and referred to as the lateral costal artery (LCA) [2]. The overall prevalence of this variant is 11–19%, with a higher prevalence in males [2, 3]. In most cases, the LCA terminates before the 4th rib. However, in 4% of individuals with the LCA variant, it extends as inferiorly as the 5th intercostal space. In bilateral cases, the vessel length often differs between sides, requiring individual assessment of each internal thoracic artery [2]. In the present case, the LCA was seen to extend to at least the 7th intercostal space.

The significance of the LCA variant has been reported in the setting of coronary artery bypass grafting (CABG) where its presence may cause a steal phenomenon when the parent ITA is used as the bypass conduit [5, 6]. Notably, males demonstrate significantly higher LCA prevalence of 22% vs 14% in females, placing them at greater risk for steal phenomenon if the vessel remains unligated during internal thoracic artery harvest [2]. This steal phenomenon can manifest as post-operative angina in which transcatheter embolization of the LCA variant was performed with relief of anginal symptoms [6]. Beyond CABG, unrecognized LCA anatomy poses a risk for intraoperative bleeding, as the vessel typically measures approximately 2 mm in caliber and can extend as far as the seventh intercostal space [1]. Prominent LCAs coursing into the lower chest or below the diaphragm can further complicate VATS procedures such as pleurectomy by introducing unexpected vascular structures in the surgical field [7]. In rare cases, the LCA variant is highlighted in the setting of thoracic outlet decompression, as documented in one case where an aberrant ITA required ligation. While this represented an adjustment to a planned surgical approach rather than a true complication, it highlights the procedural impact of these variations [8]. The present case highlights another important clinical implication of this variant where it may serve as a parasitic blood supply to a nearby pleural neoplasm or become a target of a traumatic or iatrogenic injury. Awareness of this unusual variant may aid in expeditious exploration of the bleeding source of hemothorax when the usual intercostal arteries have been ruled out.

## Data Availability

All data generated or analyzed during this study is included in this published article and its supplementary information files.

## Abbreviations

LCA: Lateral costal artery
ITA: Internal thoracic artery
CT: Computed tomography
VATS: Video-assisted thoracoscopic surgery
CABG: Coronary artery bypass grafting
NBCA: N-butyl cyanoacrylate
